# Molecular data reveal a complex population genetic structure for *Psalidodon scabripinnis* (Teleostei: Characidae) in the Atlantic Rainforest, Brazil

**DOI:** 10.1590/1678-4685-GMB-2021-0048

**Published:** 2022-02-25

**Authors:** Daniel Meneguello Limeira, Mateus Henrique Santos, Rogério Pincela Mateus, Claudete de Fátima Ruas, Mara Cristina de Almeida, Orlando Moreira, Roberto Ferreira Artoni

**Affiliations:** 1 Instituto Federal do Paraná, Londrina, PR, Brazil.; 2 Universidade Estadual de Ponta Grossa, Departamento de Biologia Estrutural Molecular e Genética, Laboratório de Genética Evolutiva, Ponta Grossa, PR, Brazil.; 3 Universidade Estadual do Centro-Oeste, Departamento de Ciências Biológicas, Laboratório de Genética e Evolução, Guarapuava, PR, Brazil.; 4 Universidade Estadual de Londrina, Departamento de Biologia Geral, Laboratório de Marcadores Moleculares e Citogenética de Plantas, Londrina, PR, Brazil.; 5 Universidade Federal de São Carlos, Departamento de Genética e Evolução, Laboratório de Citogenética e Biodiversidade, São Carlos, SP, Brazil.

**Keywords:** Lambaris, Campos do Jordão plateau, genetic diversity, microsatellites, mitochondrial data

## Abstract

Recently renamed, *Psalidodon scabripinnis* populations of Serra da Mantiqueira, previously known as *Astyanax scabripinnis* have been deeply studied in the last years. These populations are small and isolated and occur very close to the watershed between Paraíba do Sul River basin and Upper Paraná River basin, in Serra da Mantiqueira region in the Atlantic Rainforest. These conditions arouse the interest in knowing theor genetic conservation status and how they responded to the separation between the two rivers basins. Therefore, we accessed the genetic diversity of five *P. scabripinnis* populations of this region with microsatellites and mitochondrial data. The results showed a complex structure pattern that doesn’t match the simple basin separation and a reasonably conservation status when compared with other populations of the same family or with similar natural history.

## Introduction

Serra da Mantiqueira (SM) is a mountain range formed by crystalline rocks, located at Atlantic Rainflorest in southeastern Brazil. In general, its origin is associated with tectonic movements of the lower Cretaceous plate some 70 million years before the present time (Modenesi-Gauttieri *et al*., 2002). However, there is evidence of more recent tectonic activities, causing the final elevation of the SM during the Quaternary, a period that started 1.8 million years ago and extends into the present (Hiruma *et al*., 2001). Along the SM, there are flat landscapes from ancient erosions, such as the Campos do Jordão plateau, between the states of São Paulo and Minas Gerais, and Itatiaia, in the state of Rio de Janeiro (Modenesi-Gauttieri *et al*., 2002).

The Campos do Jordão plateau occurs at altitudes that reach more than 2,000 meters (Almeida and Carneiro, 1998). It is limited by steep cliffs that rise approximately 1,500 meters from the Paraíba do Sul valley (Modenesi-Gauttieri and Hiruma, 2004), consisting of transient faults directed to the northwest: the Jundiuvira Fault and the Paiol Grande Fault (Hasui *et al*., 1978).

Ingênito and Buckup (2007) investigated the potential of the SM as a biogeographic barrier for ichthyofauna between the Paraíba do Sul and Upper Paraná River basins. The authors found the coexistence of species in both basins, including one from the group *Astyanax scabripinnis* (Characiformes), identified is these work as *Astyanax* sp.1, a species now renamed *Psalidodon scabripinnis* by Terán *et al*. (2020). The authors concluded that the SM acts as an efficient barrier between the faunas of both basins. However, the work did not have a population approach and variations at this level are still open to question.

The ichthyofauna of the Paraíba do Sul River has also been characterized and studied by other authors who identified endemic regions, species introduction, and some possible headwater capture points in the basin (Bizerril, 1998). 

The *Astyanax scabripinnis* complex (Moreira-Filho and Bertollo, 1991), or *Psalidodon scabripinnis* as we will call it here, is particularly interesting because it forms isolated populations at the headwaters of streams that present great karyotype variation, wide geographical distribution, from Espírito Santo to Rio Grande do Sul (reviewed in Moreira-Filho *et al*., 2004).

Although studies are being performed in this species with chromosomal markers approximately for more than four decades, the populations of *P. scabripinnis* from the Serra da Mantiqueira have never been studied with molecular markers of diversity. Thus, our objective in this work was to access the genetic diversity of these populations with molecular markers, to test hypotheses of structuring and genetic conservation.

## Material and Methods

### Collection area

**Figure 1 -:**
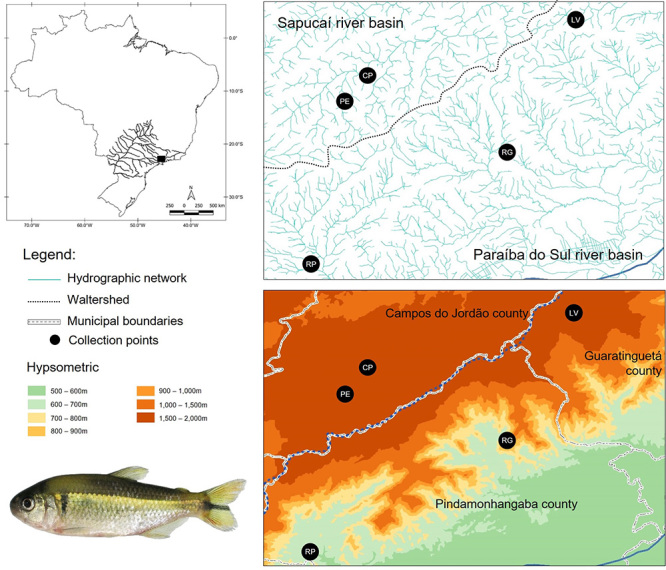
Collection sites for the examples of *Psalidodon scabripinnis*. Above and to the left, the Paraná River and Paraíba do Sul River basins; the black dot represents the collection area. Below and to the left, a specimen of *Psalidodon scabripinnis*. Above and to the right, approximation to the collection points and the hydrographic network; below and to the right, approximation of the collection sites, showing the altitude of each site.

We collected 148 specimens of *Psalidodon scabripinnis* from five different collection sites (Figure 1), each one of a different stream, three belonging to the Paraíba do Sul River basin (Córrego Lavrinhas, Ribeirão Grande, and Ribeirão Pequeno) and the other two belonging to the Sapucaí River basin, a tributary of Rio Grande, which in turn is a tributary of the Upper Paraná River (Córrego das Pedras and Ribeirão Perdizes). All the sampling sites belongs to the Serra da Mantiqueira region and are next or in the Campos do Jordão plateau.

Due to taxonomic uncertainties concerning the group, a sample of five individuals from each collection point was selected for molecular identification (barcode) using the cytochrome c oxidase I (COI) gene. In addition, we sent another sample from each point for confirmation to the Museum of Zoology at the State University of Londrina. The locations, codes assigned to the populations, sample number, municipalities, and geographic coordinates of the collection points are presented in Table 1. The same table also shows the number of the voucher generated by the deposit of some of the copies in ichthyology/zoology museums for the five populations. The small number of individuals collected in the Ribeirão Pequeno population made a deposit impossible. 

**Table 1 -:** Locations, population codes, municipalities, geographic coordinates of collection points, and voucher numbers (tumble number) of ichthyology/zoology museums.

Location/Population code	Municipalities	N	Geographic coordinates	Elevation	Voucher number
Córrego das Pedras / CP	Campos do Jordão/SP	36	22^o^43’33.2’’S 45^o^33’7.40’’W	1619 m	MZUEL 5655
Córrego Perdizes / PE	Campos do Jordão/SP	30	22^o^44’35.3’’S 45^o^34’11.6’’W	1638 m	NUP 17484
Lago Capivari / LC	Campos do Jordão/SP	30	22^o^43’02.8’’S 45^o^33’51.9’’W	1558 m	NUP 17486
Ribeirão Grande / RG	Pindamonhangaba/SP	21	22^o^46’57.38’’S 45^o^26’33.80’’W	921 m	MZUEL 5656
Córrego Lavrinhas / LV	Guaratinguetá/SP	35	22^o^40’46.8’’S 45^o^23’33.2’’W	1863 m	NUP 17482
Ribeirão Pequeno / RP	Pindamonhangaba /SP	26	22^o^52’13.75”S 45^o^35’43.51”W	606 m	

MZUEL: Zoology Museum of the State University of Londrina; NUP: Ichthyological Collection of the Limnology, Ichthyology and Aquaculture Research Group (Nupelia) of the State University of Maringá.

### Microsatellites

The total DNA was extracted using the CTAB method (Cetyltrimethylammonium bromide) (Boyce *et al*., 1989). We selected six polymorphic microsatellite loci (Table S1) described by Zaganini *et al*. (2012) to *Psalidodon (~Astyanax) altiparanae* (Characiformes), whose transferability has already been tested to *P. scabripinnis* (Limeira *et al*., 2019). The amplifications were made using the GoTaq™ Green Master Mix Kit from Promega, with forward primers plus M13 tail and with the universal primer M13 (Schuelke, 2000) marked with FAM, HEX, and NED. PCRs were performed in a final volume of 10 µl composed of 4.5 µl of the Master Mix Kit, 0.32 µl of the reverse primers and M13 (final concentration in the reaction of 0.32 pmol/µl), 0.08 µl of the forward primer (final concentration in the reaction of 0.08 pmol/µl) plus the M13 tail, 2 µl of the DNA sample (20 ng in the final reaction) and 2.78 µl of ultrapure water. The amplification cycles started with a five-minute denaturation stage at 95 ^o^C, followed by 35 cycles of 30 s at 95 ^o^C for denaturation, 30 s of annealing, where the temperature depended on the primers, and 30 s of extension at 72 ^o^C. At the end of the 35 cycles, eight more followed to mark the fragments, composed of an initial denaturation stage of 95 ^o^C, annealing at 53 ^o^C and extension at 72 ^o^C, all for 30 s. Finally, an extension stage of 72 ^o^C was completed for 5 min.

Genotyping was performed in an ABI 3500 XL sequencer (Applied Biosystems), using the GeneScan 600 LIZ™ (ThermoFisher Scientific) as the size standard and the electropherograms were interpreted using the program GELQUEST (Sequentix - Digital DNA Processing - Klein Raden, Germany), with the alleles being named for their size. Subsequently, the software TANDEM (Matschiner and Salzburger, 2009) was used for the final adjustment of the alleles.

We investigated genotyping problems, such as the existence of null alleles, stuttering, and allelic dropouts using MICRO-CHECKER 2.2.3 (Van Oosterhout *et al*., 2004). As the presence of null alleles was detected in several loci, the genotypes were adjusted using the same program. All the following analyses were conducted with the adjusted genotype matrix and with the genotype matrix without the MICRO-CHECKER adjustment and there were no significant changes in the results (data not shown). In this way, we produced the data presented here with the adjusted genotypes, with exception of F_ST_ values. Subsequently, to investigate the presence of linked loci, we tested the Linkage disequilibrium in two programs that showed similar results: ARLEQUIN 3.5.2 (Excoffier and Lischer, 2010) and GENEPOP 1.2-on line (Raymond and Rousset, 1995) and corrected with the Bonferroni Sequential Adjustment (Rice, 1989). Both tests were conducted with 10,000 dememorizations, 1,000 batches*,* and 10,000 interactions per batch.

To correct the F_ST_, we used FREENA program, with the ENA method (excluding null alleles, Chapuis and Estoup, 2007). This method corrects the positive bias induced by the presence of null alleles and corrects the F_ST_ values.

For the six loci used, the mean number of alleles per locus (N_A_), the effective number of alleles (N_E_), the number of private alleles (N_AP_), the levels of observed and expected heterozygosity (H_O_ and H_E_, respectively), and the gene fixation indices for each population were calculated using GENALEX 6.501 (Peakall and Smouse, 2012). Allelic richness (A_R_) was calculated using the HP-Rare program (Kalinowski, 2005), using the rarefaction factor equal to 20, which is the largest number of alleles found in a single locus. This program works using the rarefaction model developed by Hurlbert (1971), correcting sampling errors, since collections did not always result in the same number of individuals. 

The investigation of loci outside the Hardy-Weinberg Equilibrium was carried out using the exact tests of GENEPOP 1.2-on line (Raymond and Rousset, 1995) and ARLEQUIN 3.5.2 (Excoffier and Lischer, 2010), with 10,000 dememorizations, 1,000 batches, and 10,000 interactions per batch, as parameters for Markov chains. Both programs are based on the algorithm developed by Guo and Thompson (1992), suitable for studying populations with microsatellites that present several alleles per *locus.*

The patterns of population structure were investigated using STRUCTURE 2.3.4 (Pritchard *et al*. 2000), considering the model of admixture ancestry with allelic frequencies correlated between populations. The best K value (number of populations or clusters) was determined by testing K values from 1 to 7, with 20 replicates each. The burn-in period was 10,000 with a number of Monte Carlo simulations via Markov chains of 100,000 after the burn-in*.* The K value that best explains the structuring of populations was determined according to Evanno *et al*. (2005), using the on line tool Structure Harvester (Earl and Vonholdt, 2012). To deepen the investigation on the structuring, we conducted an analysis in STRUCTURE following the method proposed by Coulon *et al*. (2008). By this approach, initially the number of genetic groups was determined (best K value). After determination of this value, we separated the populations, according to the groups obtained and carried out new tests in each group, following the same parameters. Thus, we repeated these procedures until each test resulted in the least inclusive levels possible, that is, until the analyses did not demonstrate the hierarchization in genetic groups, superior to the populations.

The analysis of molecular variance (AMOVA) was performed in ARLEQUIN 3.5.2 (Excoffier and Lischer, 2010), with 10,000 permutations, assuming two patterns of hierarchization. We supposed initially that the hierarchization pattern follows the logic of separating both populations in two hydrographic basins: populations of water bodies that drain into the Sapucaí River basin and populations of water bodies that drain into the Paraíba do Sul River basin. In the second, we hierarchized the data assuming the formation of three groups. The first formed by the PE, RG, and LV populations, the second group formed only by the CP population, and the third by the RP population. This hierarchization hypothesis was extracted from the results of the STRUCTURE Bayesian analysis.

We investigated gene flow patterns and calculated the Effective Population Size (Ne) for each population using MIGRATE 3.6.11 (Beerli and Felsenstein, 2001;Beerli, 2006). This program is able to estimate both parameters using Bayesian inference and the principle of maximum likelihood. MIGRATE calculates two population parameters scaled by the mutation rate: the migration rate, expressed by M, and the effective population size, expressed by Ne. The rate of migration escalated by the rate of mutation, then, depends on two factors. The first, *m*, is the migration rate, which is the fraction of individuals in a population made up of immigrants or the probability that a randomly chosen individual is an immigrant and the second is, of course, the mutation rate (µ). Thus, M_i→j_= *m*_*i→j*_ /µ represents the rate of immigrants, within the effective population size, in the population *j* coming from the population *i*. The value of the mutation rate used here was 5.56 x 10^-4^ (Yue *et al*., 2007; Bradic *et al*., 2012).

To try to identify the gene flow patterns, a chain with 500,000 steps without replicates was used, being sampled every 100 steps, the first 10,000 steps being discarded (burn-in period), and single-step mutations model as parameters for the Bayesian inference. For these analyses, we used three strategies: (a) investigation of the gene flow only among the populations in the Sapucaí basin; (b) investigation of gene flow only among populations in the Paraíba do Sul basin; and (c) investigation of gene flow between the Sapucaí basin and between the Paraíba do Sul basin. In all analyses, the geographical distances between the collection points were used.

### Mitochondrial data

To obtain markers based on COI and ATPase 6/8 sequences, we used five individuals from each population, always with forward and reverse sequences. For COI, the amplifications occurred using the primers FishF1 (5’-TCAACCAACCACAAAGACATTGGCAC-3´) and FishR1 (5’-TAGACTTCTGGG TGGCCAAAGAATCA-3’) (Ward *et al*., 2005). The amplification reactions were carried out in solutions containing 2.5 mM MgCl_2_, 0.2 mM dNTPs, 0.2 µM of each primer (Forward and Reverse), and 0.2 unit of Taq DNA Polymerase and 25 ng of DNA from each individual, supplemented with 2.5 µl of enzyme buffer (10X) and ultrapure water for a final volume of 25 µl. The amplification cycles occurred with an initial denaturation of 95 ^o^C for two minutes, followed by 35 cycles of 30 s at 94 ^o^C for denaturation, 30 s at 54 ^o^C for the annealing of primers and one minute at 72 ^o^C for extension. After the cycles, a final extension was performed of 72 ^o^C for 5 min. For ATPase 6/8), the primers L8331 (5’-AAGCRTYRGCCTTTTAAGC-3’) and H9236 (5’-GTTAGTGGTCAKGGGCTTGGRTC-3’) (Sivasundar *et al*., 2001) were used. The amplification reactions were carried out under the same conditions used for COI, with the temperature of 57 ^o^C for the annealing of primers as the only alteration.

All reaction products were applied on 1% agarose gel to verify the quality of the amplifications. These products were then purified with the GFX™ PCR DNA and Gel Band Purification Kit (GE Healthcare) according to the manufacturer’s instructions. After the purifications, the COI amplified fragments were sent for sequencing in ACTGene, linked to Ludwig Biotecnologia Ltda, located in Alvorada-RS-Brazil. ATPase fragments were sent to Macrogen, Seoul-KOR.

After obtaining the sequences, we compared each individual one with deposited sequences in GenBank, to confirm similarity. Subsequently, we checked for the presence of gaps or misaligned bases in the MEGA 7.0 program (Kumar *et al*., 2015). In the same program, the forward and reverse sequences of each individual were manually corrected, that is, each mutation or absence of base determination was checked individually and the consensus sequences for each individual were generated. With the consensus sequences of each individual, all sequences of all individuals were aligned using MUSCLE (Edgar, 2004 - implemented in MEGA 7) and the mutation points and/or points of absence of base determination were checked again, one by one. Finally, all sequences obtained were deposited in NCBI GeneBank, with accession number SUB8903994 (COI) and MW628613 to MW628634 (ATPase). 

We conducted in ARLEQUIN 3.5.2 (Excoffier and Lischer, 2010) an investigation of the genetic diversity parameters for the COI and ATPase fragments: haplotypic diversity (*h*), and nucleotide diversity (π) and analysis of population structure by AMOVA (Molecular Variance Analysis). Also in ARLEQUIN, we carried out selective neutrality tests D of Tajima (Tajima, 1989) and Fs of Fu (Fu and Li, 1993) for both the COI and the ATPase.

## Results

### DNA *Barcoding*

The COI test using the mitochondrial cytochrome c oxidase I gene revealed that the genetic distance between two populations varied from 0 to 0.6%, so that all the populations studied here can be treated as belonging to the same species.

### Microsatellites analysis

The analysis with the MICRO-CHECKER revealed the presence of stutter only in *locus* 12 of the population of Córrego Lavrinhas. Allelic dropouts were not observed. Null alleles were visualized in several loci. The Linkage disequilibrium analysis, after the Bonferroni Sequential Adjustment (Rice, 1989), did not result in significant values between at least two of the loci in all populations and, therefore, no locus was removed from the analysis.

After adjusting the genotypes, the mean number of alleles, for all loci in all populations, ranged from 4.833 to 9.833, the mean number of effective alleles ranged from 3.443 to 6.447, and the mean allelic richness from 4.591 to 7.785. The means of observed and expected heterozygosities, in all populations, ranged from 0.563 to 0.698 and 0.665 to 0.825, respectively (Table 2). The population of Córrego das Pedras (CP) showed the highest values of allelic diversity parameters (number of alleles, effective number of alleles, and allelic richness), and the least allelic diversity was presented by the PE population. 

**Table 2 -:** Genetic diversity parameters of five *Psalidodon scabripinnis* populations in the Sapucaí and Paraíba do Sul River basins, Serra da Mantiqueira region.

	Pop	Locus	N	N_A_	N_E_	A_R_	N_AP_	H_O_	H_E_	F_IS_	
Sapucaí-Grande-Paraná River Basin
	CP	Asty21	30	12	6.977	7.662	6	0.733	0.857	0.144	^*^
		Asty23	33	11	4.829	7.268	2	0.727	0.793	0.083	
		Asty26	26	5	4.147	4.290	-	0.769	0.759	-0.014	
		Asty27	15	7	5.488	5.707	2	0.600	0.818	0.266	^*^
		Asty12	16	7	5.12	5.970	2	0.688	0.805	0.146	
		Asty04	20	17	12.121	9.941	13	0.5	0.918	0.455	^*^
		M/Ƹ		9.833	6.447	6.806	25	0.670	0.825	0.180	
	PE	Asty21	23	7	4.921	6.493	-	0.696	0.797	0.127	
		Asty23	30	4	2.975	3.557	-	0.767	0.664	-0.155	
		Asty26	30	4	2.507	3.326	-	0.467	0.601	0.224	
		Asty27	11	4	3.227	3.333	-	0.545	0.690	0.210	^*^
		Asty12	23	5	3.752	4.621	-	0.696	0.733	0.052	
		Asty04	13	5	3.756	4.824	-	0.615	0.734	0.161	
		M/Ƹ		4.833	3.523	4.359	-	0.631	0.703	0.103	
Paraíba do Sul River Basin
	RG	Asty21	21	6	4.594	5.379	-	0.81	0.782	-0.035	
		Asty23	21	5	3.279	3.952	-	0.714	0.695	-0.028	
		Asty26	21	4	2.602	3.801	-	0.524	0.616	0.149	
		Asty27	5	4	3.333	3.598	1	0.2	0.7	0.714	
		Asty12	17	6	4.587	5.458	-	0.647	0.782	0.173	
		Asty04	13	5	3.756	4.666	-	0.538	0.734	0.266	
		M/Ƹ		5	3.692	4.476	1	0.572	0.718	0.207	
	LV	Asty21	26	9	6.259	7.128	-	0.692	0.84	0.176	
		Asty23	22	9	4.99	6.660	-	0.636	0.8	0.204	
		Asty26	35	3	1.514	2.822	-	0.286	0.34	0.159	
		Asty27	8	4	3.282	3.429	-	0.625	0.695	0.101	
		Asty12	13	5	3.314	4.479	-	0.615	0.698	0.119	
		Asty04	23	5	2.612	4.002	-	0.522	0.617	0.155	
		M/Ƹ		5.833	3.662	4.753	-	0.563	0.665	0.152	
	RP	Asty21	19	5	3.455	4.459	-	0.632	0.711	0.111	
		Asty23	25	9	3.858	6.173	1	0.84	0.741	-0.134	
		Asty26	25	6	2.358	4.780	3	0.52	0.576	0.097	
		Asty27	14	5	3.469	4.044	-	0.643	0.712	0.097	
		Asty12	17	9	5.402	6.608	3	0.706	0.815	0.134	
		Asty04	26	3	2.058	2.385	1	0.846	0.514	-0.646	^*^
		M/Ƹ		6.167	3.433	4.742	8	0.698	0.678	-0.057	

N: sample size of each population, N_A_: number of different alleles; N_E_: effective number of alleles; A_R_: allelic richness; N_AP_: number of private alleles; H_O_: observed heterozygosity; H_E_: expected heterozygosity; F_IS_: gene fixation index; ^*^ *loci* with significant deviations from the expected Hardy-Weinberg Equilibrium after the sequential Bonferroni adjustment, M/Ƹ: mean (for N, N_A_, N_E_, A_R_, H_O_, H_E_, and F_IS_) or sum (for N_AP_) of diversity parameters.

The highest heterozygosity was observed in the RP population (0.698), while the lowest was observed in the LV population (0.563), while the largest heterozygote deficiency was in the RG population (0.207). Gene fixation indices (F_IS_) and the numerical comparison between the expected and observed heterozygosities suggest that there is no deficit of heterozygotes only in the RP population and frequent inbreeding between populations. Only five loci among the populations (about 14%) were found outside the EHW (Table 2). 

**Figure 2 -:**
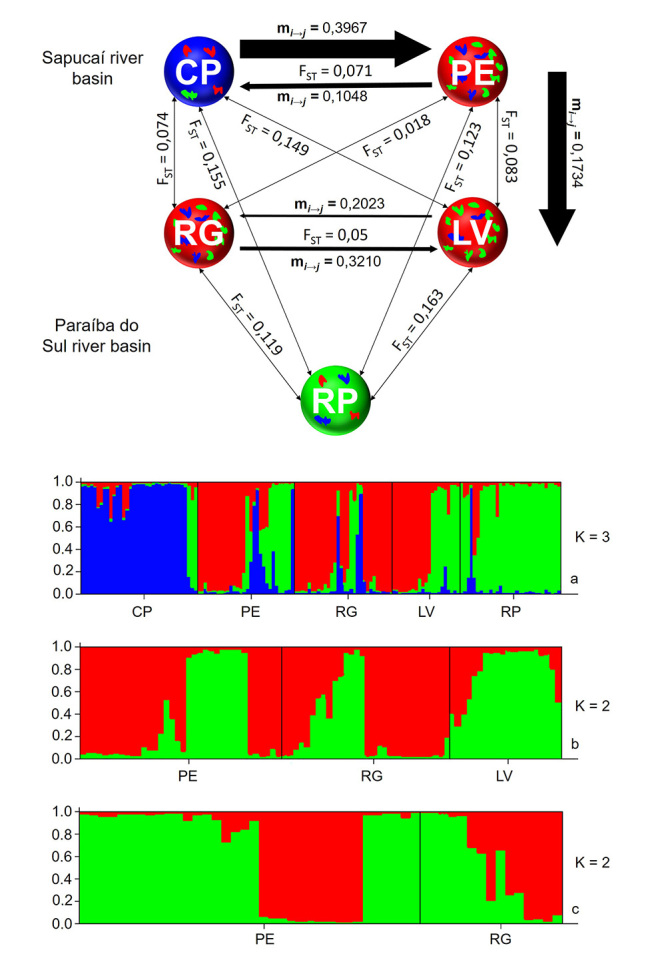
Results of the genetic structuring analysis of five *Psalidodon scabripinnis* populations in the Sapucaí and Paraíba do Sul basins. Above, paired F_ST_ (narrow arrows) and migration rate (m_i → j_, broad arrows) for the five populations. The thickness of the arrows is proportional to the migration rate. Non-significant values for the migration rate are not shown (P < 0.05, for all values of F_ST_). Below, STRUCTURE results with the three runs (a: CP+PE+RG+LV+RP; b: PE+RG+LV; c: PE+RG).

Genetic structuring via F_ST_ demonstrated that, genetically, RP differs the most from the others, followed by CP. The PE, RG, and LV populations have little genetic differentiation between them. The global F_ST_ value was 0.098, the F_ST_ values pair by pair are shown in Figure 2 (P<0.05 for all values). The first Bayesian analysis test of STRUCTURE points in the same direction, indicating three genetic groups (K=3), with CP and RP being markedly different from the other populations, while the other three populations do not demonstrate structuring among them. We noted that LV, PE, and RG contain alleles of the same founders groups from CP and RP less frequently and of another group, not investigated here, more frequently (Figure 2a). A new test was carried out, at this new hierarchical level, with the three populations of the second group, resulting in K=2. PE and RG are composed of both origins, whereas LV is basically composed of only one (Figure 2b). In the last run, only with the PE and RG populations and assuming K=2, it was noted that there is no evident structure between these two populations (Figure 2c).

The gene flow analysis, through the rate of migrants investigated with MIGRATE, among populations in the Sapucaí River basin, showed that there is a greater gene flow between CP and PE populations, with the greatest direction of migration from PE to CP, around four times higher. In more detail, the rate of immigrants in CP from PE is almost 40%, while the rate of immigrants in the opposite direction is approximately 10% (Figure 2). Among the populations of the Paraíba do Sul River, strong evidence of gene flow was found between the RG and LV populations. The rate of immigrants in LV from RG is around 32%, while the rate of immigrants in LV from RG is 20% (Figure 2). 

The hypothesis of the formation of three groups or clusters, tested by AMOVA for both microsatellites and sequences, seems to best explain the population structure, as our data show (Table 3). This hypothesis, inferred from the results of STRUCTURE, considers a group formed by the populations PE, RG, and LV, another only by the population CP, and a third by the RP population. In this approach, the largest portion of the variation, for microsatellites, is found between populations (88.82%), followed by variation between groups (7.48%), and variation between populations within groups (3.7%). Table 3, which also shows the values of the F-statistics (F_ST_, F_SC_, and F_CT_) and their probabilities.

**Table 3 -:** Values of the genetic variation of three markers (Microsatellites, COI, and ATPase) obtained through AMOVA under two hypotheses of hierarchical data (Sapucaí and Paraíba do Sul Basins and groups - PE+LV+RG, CP, and RP).

	Hierarchization of basins				Hierarchization of groups		
	SSRs	COI	ATPase		SSRs	COI	ATPase
Between groups/basins	1.49	-7.44	-15.84		7.48	67.59	65.31
Among populations within groups/basins	8.529	53.27	67.00		3.70	-10.03	-1.17
Among populations	89.99	54.17	48.84		88.82	42.45	35.86
	Hierarchization of basins				Hierarchization of groups		
F_CT_	0.01490	-0.07441	-0.15835		0.07478	0.67585	0.65315
F_SC_	0.08649^*^	0.49583^*^	0.57839^*^		0.04005	-0.30952	-0.03383
F_ST_	0.10010^*^	0.45831^*^	0.51162^*^		0.11183^*^	0.57552^*^	0.64141^*^

The investigation of bottlenecks, assuming the two-phased mutation model (TPM), according to the Wilcoxon test (P_WT_), the most robust when there are less than 20 microsatellite loci, revealed a significant heterozygosity excess, suggesting a recent bottleneck only for the PE and RG populations (significance level of P<0.05). The probability values can be seen in Table 4, along with the effective population sizes (Ne), calculated using the Bayesian inference.

**Table 4 -:** Effective population sizes (Ne) provided by the MIGRATE program and probabilities of the Wilcoxon test (P_WT_), according to the two-phased mutation model (TPM), to test the heterozygosity excess, provided by the BOTTLENECK program, for five populations of *Psalidodon scabripinnis* from the Serra da Mantiqueira region.

	Effective population size		Heterozygosity Excess
Pop	Ne		P_WT_ (TPM)
CP	33.7		0.578125
PE	10.7		0.007813
RG	16.9		0.007813
LV	33.5		0.921875
RP	2.9		0.921875

### Mitochondrial DNA analysis

The amplification of the cytochrome c oxidase I (COI) gene resulted in fragments of, on average, 652 bp, whereas the amplifications for the ATPase gene, resulted in fragments of, on average, 884 bp, after the alignment and editing of the sequences.

The values of haplotypic and nucleotide diversity for the ATPase gene ranged from 0 to 0.8333 and from 0 to 0.0073, respectively. As for the COI values, they ranged from 0 to 0.4 and from 0 to 0.37, respectively. No significant values were obtained for the majority of neutrality tests (D for Tajima and Fs for Fu). The results of the neutrality tests, as well as the diversity values, are shown in Table 5.

**Table 5 -:** Values of genetic diversity and selective neutrality test of ATPase and COI for five populations of *Psalidodon scabripinnis* from the Serra da Mantiqueira region.

	ATPase						COI				
Pop	*N*	*h*	π	*D*	*Fs*		*N*	*h*	π	*D*	*Fs*
CP	4	0	0	0	0		4	0	0	0	0
PE	5	0.6 0.1753	0.0007 0.0007	1.2247	0.6262		5	0	0	0	0
LV	4	0	0	0	0		3	0	0	0	0
RP	4	0.8333 0.2224	0.0046 0.0035	-0.4464	1.2253		3	0.4 0.2373	0.0037 0.0029	-1.1455	3.0225
RG	5	0.8 0.1640	0.0013 0.0012	0.2431	-0.4754		5	0	0	0	0

N: sample size; *h*: haplotypic diversity above and below, in the same cell the standard deviation; π: nucleotide diversity above and below, in the same cell the standard deviation; *D:* value of the D test of Tajima; *Fs:* value of the Fs test of Fu.

The results of the AMOVA for the mitochondrial markers (Table 3), showed that the largest part of the variation was found between the groups (67.59% for COI and 65.31% for ATPase), followed by variation between populations (42.45% for COI and 35.86% for ATPase), and variation between populations within groups (-10.03% for COI and -1.17% for ATPase).

## Discussion

Although the Serra da Mantiqueira has proven to be an efficient barrier to ichthyofauna (Ingenito and Buckup, 2007), our data demonstrated that the structuring of *P. scabripinnis* populations on the Campos do Jordão plateau does not follow the division of the basins. Thus, the simple hypothesis of the hierarchy of the populations of *P. scabripinnis* in the Serra da Mantiqueira in two groups equivalent to the basins of Sapucaí-Grande-Alto Paraná and Paraíba do Sul, does not seem to explain the genetic variation between them. This is indicated by the results of the Bayesian inference for the investigation of population structure, carried out by the STRUCTURE program. The methodology suggested by Coulon *et al*. (2008) was effective in showing the structure of populations, clarifying the differences between them. Note that the RP and CP populations are more genetically distant from the group formed by PE+RG+LV, making it necessary to formulate an alternative hypothesis to explain the genetic variation of these populations.

Similarly, the hypothesis of hierarchization in two groups formed by the two basins, tested by AMOVA, both for microsatellite markers and for mitochondrial DNA, was not able to satisfactorily explain the variation. The variation can be better explained by hierarchizing the data into groups (RP, CP, and PE+RG+LV), as suggested by the Bayesian analysis of Structure. The analysis of the AMOVA results of both markers (Table 3) also suggests that the structuring of the populations is recent, since only the microsatellites demonstrated clear structuring.

The values and F_ST_ point in the same direction as the AMOVA and Bayesian inference of STRUTURE. Values above the global mean (F_ST_=0.098) are found between RP and any other of the four populations and between CP and LV, populations from the same basin (Figure 2). The lowest value occurs between RG and PE, populations found in different basins. Even disregarding the hierarchy, the F_ST_ values also demonstrated a marked differentiation between populations. In order to compare these differences, we can mention studies with *Astyanax mexicanus*, where the highest F_ST_ value between two populations was 0.51 and the lowest 0.01 (Bradic *et al*., 2012). 

The use of AMOVA has been shown to be efficient for low migratory fish. Bradic *et al*. (2012) tested several hypotheses of population structure for *A. mexicanus,* inferred from the allelic diversity and the STRUCTURE data. The hypothesis that best explained the hierarchy of the data did not group the populations simply into cave or surface areas, demonstrating great biogeographic complexity, a scenario similar to that found in the current work. In populations with greater geographic separation and barriers more difficult to cross, the structuring hypotheses can be inferred directly from biogeographic data. For example, populations of the killifish *Aphanius fasciatus* collected in Greece and Turkey and separated by the Aegean Sea, presented genetic structure compatible with the geographic distribution (Triantafyllidis *et al*., 2007).

In fact, there is ample evidence of recent tectonic events in the region known as the Campos do Jordão Plateau. Hiruma *et al*. (2001) reported several headwaters capture events that occurred recently (about 10,000 years ago) in the Campos do Jordão plateau region. Modenesi-Gauttieri *et al*. (2002) identified drainage captures at the headwaters of Ribeirão do Sino and Córrego Lavrinhas, also found evidence of capture at several other drainages in the Campos do Jordão plateau. Evidence of recent tectonism, as well as drainage captures, reinforces the hypothesis that vicariance events among the sampled populations did not occur simultaneously and may have preceded headwaters captures.

The migration rates identified by MIGRATE help us to understand this scenario. There is strong evidence of gene flow from PE to CP while reverse flow is unlikely. These findings are reinforced by the reasonable number of loci outside the Hardy-Weinberg equilibrium observed in CP. In the Paraíba do Sul basin, there is great evidence of gene flow from RG to LV and moderate to the inverse. The RP population presented the greatest geographical distance from other populations, being 606 meters in height, with an elevation of approximately 1,000 meters less than the other populations. It is possible that, during the uplift of the crystalline edges, it was the first to separate itself from the other populations, remaining isolated for a longer time (Ingenito and Buckup, 2007)

The comparison between geographic distances and the F_ST_ reinforces the explanation above. Within the same basin, the highest values of F_ST_ paired between RP and another population also correspond to the greatest geographical distances. 

After RP, CP is the most genetically different population from the rest. Among the populations of the Sapucaí basin, CP is the furthest from the watershed and the one with the lowest altitude. As with RP, it is likely that this population has been isolated longer than PE from the populations of the Sapucaí basin. Although there are signs of gene flow from PE to CP, with weak signs to the contrary, there is a distinct difference between them. The flow from PE to CP can be explained by the geographical proximity between them. In addition, the higher PE altitude in relation to RP may contribute to the migration patterns seen. 

Following this reasoning, the same applies to LV, which is also more distant from the populations of the Sapucaí basin and, among those in the Paraíba do Sul basin, is the one that lives at the highest altitude. These values indicate that LV may have been the second to separate from other populations and, due to the proximity to RG, still maintains migrations. However, the difference in altitude between RG and LV makes it difficult to explain the migration patterns indicated by MIGRATE, since there was more evidence of migration from RG to LV than to the contrary. The fact that LV does not have private alleles corroborates this observation. As RG is at 921 m in relation to LV at 1863 meters of altitude, it is not parsimonious to assume that there is easier migration from the lowest to the highest location than the other way around. However, many other factors, in addition to the altitude, that were not the subject of this study, can influence migration patterns (Fausch *et al*., 2002). According to Jonsson and Jonsson (1993), individuals from the same population exhibit different migratory behaviors, for example, migrants and residents may have different rates of growth and fertility. Bradic *et al*. (2012) showed, in *Astyanax mexicanus,* that cave populations receive more alleles from surface populations than they donate. The incursions of fish from the surface to the caves can occur for several reasons, including the rain regime. However, the authors also noted that some cave populations migrate more than others. Migration patterns may be contributing to the maintenance of genetic variation among populations in the region.

The populations of *P. scabripinnis* from the Serra da Mantiqueira had not yet been studied at the molecular level. Similarly, little was known about their genetic conservation status. Studies that accessed the genetic variability of *Psalidodon* (~*Astyanax*) populations with microsatellite data are extremely rare, despite of the large number of studies with with chromosomal markers (Souza and Moreira-Filho, 1995; Néo *et al*., 2000; Moreira-Filho *et al*., 2004; Castro *et al*., 2014). As an example, we find the populations of *Astyanax mexicanus*, which inhabit surface waters and caves, forming small groups with low gene flow (Protas *et al*., 2006), having, therefore, a natural history similar to the populations of *P. scabripinnis*. Panaram and Browsky (2005) analyzed populations of *A. mexicanus* with data from microsatellites and observed that the heterozygosity of populations that they classified as small ranged from 0 to 0.254, while in large populations it ranged from 0.139 to 0.656. Other studies with the same species revealed populations with enormous variation in the levels of heterozygosity observed: 0 to 0.905 (Strecker *et al*., 2003; Bradic *et al*., 2012). Zaganini *et al*. (2012) obtained for *Psalidodon (~Astyanax) altiparanae* in the Upper Paraná River basin high levels of heterozygosity ranging from 0.5 to 0.852, and high levels of gene fixation in almost all *loci*.

When comparing the values of observed heterozygosity of the populations of *P. scabripinnis* studied here, ranging from 0.563 to 0.698, with the studies mentioned above, it is noted that they are relatively high and less variable than the heterozygosities of populations of *A. mexicanus*. It is interesting to note here that the majority of the populations in the studies cited and our results showed a deficit of heterozygotes (Table 2).

The mean number of alleles obtained here varied from 4.8 to 9.8, while in other *Psalidodon* (*~Astyanax*) the numbers varied from 2.23 to 15, as in *A. mexicanus* (Strecker *et al*., 2003; Bradic *et al*., 2012). For a population of *P. altiparanae,* the mean was 7.5 (Zaganini *et al*., 2012). Although some of the works with *Astyanax* mention the number of alleles, but not allelic richness, the latter is a more informative parameter for comparing the diversity between populations, since it applies a rarefaction factor (revised by Foulley and Ollivier, 2006). This comparison demonstrates the values obtained herein (4.359 to 6.806) are within the variation found for the genus *Psalidodon* or *Astyanax*. An example of this is seen for the variation between the allelic richness means from 1.21 to 5.21 in *A. mexicanus* (Panaram and Borowski, 2005; Bradic *et al*., 2012).

The lack of studies with population-based approaches on the *Psalidodon* genus (~ *Astyanax*), makes comparisons difficult, and restricts the power of broader inferences. However, there is a greater variation in several parameters of *A. mexicanus*, such as levels of heterozygosity and allelic richness. This may be due to the large number of existing and studied populations of this species, which enabled description of the colonization of caves from various origins and in several different times (Bradic *et al*., 2012). This complex biogeographic scenario tends to overestimate the variation in the parameters of genetic diversity, to the detriment of the populations analyzed here, which are more restricted. However, the genetic variation of *A. mexicanus* is expected, since Central America is considered the center of origin of *Astyanax* (Ornelas-Garcia, 2011).

Among the populations studied herein, only RP does not present a deficit of heterozygotes (F_IS_ = -0.057). The subdivision of populations can lead to an increase in inbreeding, which would, consequently, increase homozygosity and reduce heterozygosity. Reductions in effective population size are often linked to population genetic bottlenecks. In this scenario, genetic diversity tends to decrease, leading to an increase in inbreeding and decreasing the viability of populations (Spencer *et al*., 2000). According to Cornuet and Luikart (1996), during the bottleneck, allelic diversity, measured through the allelic richness and number of alleles, is initially affected due to the loss of alleles, causing the so-called allelic deficiency. Thus, the observed allelic diversity is less than the expected diversity in relation to the observed heterozygosity, a phenomenon known as heterozygosity excess. The heterozygosity excess is identified according to Nei (1987), while the excess of heterozygotes is calculated according to the Hardy-Weinberg Equilibrium (Cornuet and Luikart, 1996). The BOTTLENECK program showed evidence of heterozygosity excess for the PE and RG populations (P <0.05). These also presented small effective population sizes (10.7 and 16.9, respectively, the two smallest among the five populations, disregarding RP) and the two smallest allelic diversity values.

Comparing PE and RG with the other populations of *P. scabripinnis* in this work and with the similar populations mentioned above, it can be noted that they have low levels of allelic diversity (number of alleles, effective number of alleles, and allelic richness) and indications of inbreeding (F_IS_ = 0.103 and 0.207, respectively). Similarly, mitochondrial DNA data indicate that these two populations have low levels of both haplotypic and nucleotide diversity for the COI gene and moderate for ATPase. The set of these factors, together with the small effective population size (Ne), are indications that both populations went through bottlenecks. According to Cornuet and Luikart (1996), when a population enters a genetic bottleneck, caused by the reduction in the effective population size, inbreeding leads, initially, to the loss of allelic diversity. What is expected in the next steps, is that a heterozygosity excess appears (according to Nei, 1987), that is, the observed number of alleles is less than would be expected for the observed heterozygosity. Finally, heterozygosity is expected to decrease. Thus, it can be inferred that the bottleneck events of PE and RG are recent, as they still present moderate levels of observed heterozygosity. Among the populations in the current work, others also have relatively high levels of inbreeding or low effective population sizes, respectively CP and RP, for example. However, the allelic diversity in these populations is high which is probably why they did not show signs of bottleneck*.*

In fish, the decrease in population sizes can be better detected in species with high commercial interest, as well as which the genetic effects of this decrease. Vera *et al*. (2010) studied six populations of trout (*Salmo truta caspius -* Salmoniforms: Salmonidae) and detected a population bottleneck for one of them. This population also presented low genetic diversity, which, according to the authors, may have been caused by overexploitation of fishing, pollution, or destruction of spawning sites. Ferreira *et al*. (2016) detected signs of bottleneck in five of six populations of *Geophagus braziliensis* studied, and the only population that did not present a bottleneck is the one in the least anthropized environment.

Our data showed that the genetic structure of *P. scabripinnis* populations in the Serra da Mantiqueira is complex and must have been influenced by different geological events in a non-simultaneous manner. Although they are small and isolated, the populations living there present good levels of genetic variation, when compared to similar populations or those with similar biology.

## Supplementary material

Table S1 - Microsatellites annealing temperatures used in gradient test, repeat motifs and alleles length used in population study of six *Psalidodon scabripinnis* populations from the Serra da Mantiqueira region.
