# Supplementary material for: Molecular data reveal a complex population genetic structure for Psalidodon scabripinnis (Teleostei: Characidae) in the Atlantic Rainforest, Brazil
Source: Genet Mol Biol. 2022 Feb 25;45(1):e20210048. doi: 10.1590/1678-4685-GMB-2021-0048 (PMC8892460; doi:10.1590/1678-4685-GMB-2021-0048)
Supplement: Table S1 - [file 1415-4757-GMB-45-1-e20210048-s1.pdf]

Genetics and Molecular Biology
DOI: https://doi.org/10.1590/1678-4685-GMB-2021-0048

Supplementary Material to "Molecular data reveal a complex population genetic structure for *Psalidodon scabripinnis* (Teleostei: Characidae) in the Atlantic Rainforest, Brazil"

Table S1 – Microsatellites annealing temperatures used in gradient test, repeat motifs and alleles length used in population study of six *Psalidodon scabripinnis* populations from the Serra da Mantiqueira region.

Primers	T°C annealing	Repeat motifs*	Allele size*
Asty 04	56 °C	$(AC)_{17}$	200
Asty 12	58 °C	$(GT)_{8}$	163
Asty 21	54 °C	$(CA)_{9}$	150
Asty 23	54 °C	$(CA)_{12}$	160
Asty 26	54 °C	$(GT)_{8}$	190
Asty 27	58 °C	$(GT)_{8}$	150
